# What is the optimal surgical treatment for Neer type IIB (IIC) distal clavicle fractures? A systematic review and meta-analysis

**DOI:** 10.1186/s13018-022-03108-2

**Published:** 2022-04-07

**Authors:** Andreas Panagopoulos, Konstantina Solou, Irini Tatani, Ioannis K. Triantafyllopoulos, John Lakoumentas, Antonis Kouzelis, Vasileios Athanasiou, Zinon T. Kokkalis

**Affiliations:** 1 Department of Shoulder and Elbow Surgery, Patras University Hospital, Papanikolaou 1, 26504 Rio-Patras, Greece; 2 Hygeia Private Hospital, Athens, Greece; 3 Department of Medical Physics, School of Medicine, Patras University, Rio-Patras, Greece

**Keywords:** Distal clavicle fractures, Neer type IIB, Coracoclavicular stabilization, Hook plate, Locking plate, ACJ transfixation

## Abstract

**Background:**

The purpose of the present study was to systematically review the current treatment strategies for the treatment of Neer type IIB distal clavicle fractures in terms of functional outcome and complication rates and to examine the most appropriate surgical method by comparing all the available surgical techniques and implants.

**Methods:**

We performed a systematic review of the existing literature (2000–2021) in accordance with the PRISMA statement. We searched PubMed, Scopus, Web of Science, Research Gate and Google Scholar using the general terms ‘distal AND clavicle AND fracture’ to capture as many reports as possible. The MINORS tool was used to assess the risk of bias of the nonrandomized studies. We categorized the reported surgical techniques into four main types: open or arthroscopic coracoclavicular (CC) stabilization, locking plate fixation with or without CC augmentation, hook plate fixation and acromioclavicular joint (ACJ) transfixation. We reported findings for two main outcomes: clinical results and complication rates categorized into major and minor.

**Results:**

Our database search yielded a total of 630 records; 34 studies were appropriate for qualitative analysis. There were 790 patients, with a mean age of 40.1 years, a female percentage of 37% and a mean follow-up period of 29.3 months. In total, 132 patients received a hook plate, 252 received a locking plate, 368 received CC stabilization and 41 received transacromial transfixation. All studies were retrospective and had fair MINORS scores. Locking plate, CC stabilization and ACJ transfixation showed similar clinical results but were much better than hook plate fixation; CC augmentation did not significantly improve the outcome of locking plate fixation. The rate of major complications was similar among groups; hook plate and AC joint transfixation had the worst rates of minor complications. Open CC techniques were slightly better than arthroscopic techniques.

**Conclusions:**

The present systematic review for the optimal fixation method for Neer type IIB fractures of the distal clavicle showed similar major complication rates among techniques; the hook plate technique demonstrated inferior clinical results to other techniques. Open CC stabilization and locking plate fixation without CC augmentation seem to be the best available treatment options.

## Introduction

Fractures of the distal end of the clavicle account for 10–30% of all clavicle fractures, are displaced in 50% of cases and, when treated conservatively, can lead to symptomatic malunion or nonunion in 10–44% of cases [1, 2]. Displaced fractures are considered the Neer-Graig subtypes IIA, IIB and V, which usually require operative intervention [3–5]. Cho et al. [6] proposed a modified classification system to include the tiny “extralateral” type (IIC), where both of the CC ligaments are detached from the medial fragment; this type may not be amenable for traditional hardware due to its small size, and other techniques of CC stabilization are needed [7].

To date, there is no optimal surgical technique for managing the unstable types (IIA, IIB, IIC or V) of distal clavicle fractures. Numerous surgical techniques have been described in the literature and consist of three main categories: (a) *rigid internal fixation* with plates, coracoclavicular screws and Knowles pins; (b) *flexible fixation* with or without arthroscopic assistance (tension band wiring and CC stabilization with buttons, sutures, anchors, tapes, cables and synthetic grafts, autografts or allografts); and (c) *mixed techniques*, especially in terms of coracoclavicular augmentation to support a standard locking plate using screws, anchors, buttons or cables [7–18].

In a recent survey [19] among British Elbow and Shoulder Society consultant surgeons, there was considerable heterogeneity in the management of patients with distal clavicle fractures; the most important factor in favour of operative treatment was the degree of displacement (90%), while the most common operative intervention was the locking plate (68%), although there was no clear consensus regarding other fixation methods. According to the authors, the indications for surgery and the optimal fixation method remain uncertain, indicating a clear need for pragmatic multicentre clinical research in this area.

To date, several systematic reviews and meta-analyses [20–28] have shown inconsistent clinical results, time to union and complication rates in favour of one technique to another. For example, Asadollahi and Bucknill (2019) [23], in a systematic review of eleven comparative hook plate studies, including 634 patients, found no significant difference in the functional outcomes and union rates among hook plate fixation, coracoclavicular (CC) stabilization, and locking plate fixation. In contrast, Uittenbogaard et al. (2021) [26], in a systematic review of 59 studies, including 2284 patients, found that hook plates showed lower Constant-Murley scores than coracoclavicular fixation, but there was no significant difference when the hook plate was compared with the locking plate and tension band wire/K-wire groups. An important bias of these studies is the general homogenization of all displaced fractures as Neer type ΙΙ without clarification of the specific subtypes, especially IIA and IIB (IIC). In an effort to increase the current evidence on distal clavicle fractures, we have already published [27] a systematic review on the safety and efficacy of coracoclavicular fixation techniques in Neer type IIB (IIC) fractures. We included 21 studies with 421 patients; the reported clinical results were very good to excellent, and the overall major and minor complication rates were 2.6% and 12.8%, respectively.

Based on all these controversies, the purpose of the present study was to systematically review the current treatment strategies for Neer type IIB (or IIC) distal clavicle fractures in terms of functional outcomes and complication rates and to examine the most appropriate surgical method for this particular type of fracture, comparing all the available surgical techniques and implants.

## Material and methods

We performed a systematic review of the existing literature for Neer type IIB (IIC) distal clavicle fractures in accordance with the Preferred Reporting Items for Systematic Reviews and Meta-Analyses (PRISMA) statement [29].

### Inclusion criteria

Our purpose was to identify randomized trials, cohort or case–control studies, comparative studies and case series, evaluating the clinical outcomes and complication rates of Neer type IIB (C) distal clavicle fractures in adult patients. We did not exclude comparative studies of two or more different surgical methods, but we excluded patients with other types of distal clavicle fractures (IIA, V) when presented together with type IIB fractures. Studies were only reviewed if the published manuscript was in the English, French, German or Spanish language; had at least 6 months follow-up evaluation; and provided clinical outcomes with objective clinical scores, complication rates and reoperation rates. We excluded any studies that did not meet the above criteria, studies that reported on fewer than 5 patients and comparative studies of different Neer types that did not separate the outcomes for each type.

### Study identification and selection

We searched the PubMed (Medline and PubMed Central), Scopus, Web of Science, Research Gate, Google Scholar, and Cochrane Central Register of Controlled Trials electronic databases to retrieve studies published between January 2000 and November 2021. Our search strategy included a combination of keyword terms, including ‘distal AND clavicle AND fracture’, to capture as many reports as possible using a more general terminology. We also supplemented our search by screening the references of relevant published manuscripts, and we exported all captured studies into a reference manager library (EndNote X9), removing all duplicates. The results were evaluated by two independent reviewers (AP and ZK) at both the title-abstract and full-text levels; any discrepancies were solved during title-abstract screening by including the article by default and during full-text screening by senior author consensus.

### Data collection

All relevant data were extracted using piloted forms and exported to a digital spreadsheet (Microsoft® Excel). Data extraction was performed by two independent reviewers. We classified extraction fields into 5 main categories: study methods, demographics, surgical intervention, clinical outcomes and complications. Any discrepancies in the extracted data were resolved by thoroughly evaluating the manuscripts during consensus meetings.

### Risk of bias assessment

We did not find any randomized controlled trials (RCTs); thus, the Methodological Index for Non-Randomized Studies (MINORS) was used to assess the risk of bias in nonrandomized studies [30]. For all studies, the first 8 items of this tool included the following: a clearly stated aim; the inclusion of consecutive patients; prospective data collection; endpoints appropriate for the study aim; unbiased assessment of study endpoint; < 5% lost to follow-up; and a prospective calculation of study size. Additional criteria in the case of comparative studies were an adequate control group, contemporary groups, baseline equivalence, and adequate statistical analyses. The items were scored 0 if not reported, 1 when reported but inadequate, and 2 when reported and adequate; the global ideal score was 16 for noncomparative studies and 24 for comparative studies. Methodological quality was categorized a priori as follows: a score of 0–8 or 0–12 was considered poor quality, 9–12 or 13–18 was considered fair quality, and 13–16 or 19–24 was considered excellent quality for noncomparative and comparative studies, respectively.

### Data synthesis

We synthesised all studies qualitatively using descriptive statistics where applicable. We categorized the reported surgical techniques into four main types: (a) open or arthroscopic *coracoclavicular stabilization (CCS)* with buttons, sutures, tapes, wires, cables with or without interfragmentary sutures, tension bands or KWs; (b)* locking plate fixation (LPF)* with or without CC augmentation with anchors, buttons, CC screws or sutures and/or interfragmentary fixation; (c) *hook plate fixation (HPF)* and d) *AC joint* trans*fixation (ACJTF)* using KW or Steinman pins. We reported findings for two main outcomes: (a) clinical results, evaluated by synthesising and summarizing data from objective clinical scores specifically designed for the shoulder (CS: Constant Score, ASES: American Shoulder and Elbow Surgeons Shoulder score, UCLA: University of California at Los Angeles Shoulder Score) and (b) complication rate, categorized into major (peri-implant fracture, implant failure, nonunion, coracoid or acromial fracture, deep infection) and minor (malunion—delayed union, hardware irritation/migration/prominence or breakage, subacromial osteolysis, clavicular erosion, button subsidence, peri-anchor or screw osteolysis, slight loss of reduction, AC joint arthrosis, pain-discomfort, frozen shoulder stiffness, superficial infection and hypertrophic scar-wound breakdown).

### Statistical analysis

Primarily, a set of clinical scores was available for meta-analysis among studies. The default clinical score that was used was the Constant score, available in the majority of the studies; otherwise, ASES or UCLA scores were used (with the latter one being converted to the 0–100 scale). The effect size that was meta-analysed was the mean ± SD. In cases where the range (min–max) was available instead of SD, the SD was approximately computed as the range divided by the number 4. Pooling effect sizes were held with the inverse variance method, tau^2 was computed via the Sidik-Jonkman (SJ) estimator and its confidence intervals via the Q-profile method, while Hartung-Knapp adjustment for random effects model was also used. Overall, the random effects model was utilized, and means were considered untransformed (raw). Heterogeneity was quantified with the indices I^2 and tau^2, as well as with the application of Cochran’s Q test. Confidence and prediction intervals were computed at the level of 95%. Finally, the meta-analysis outcome visualization was performed with summary forest plots.

In sequence, a comparison of the meta-analysis outcomes among sets of studies was performed with the use of an analysis of variance (ANOVA) procedure that took into account the generated summary statistics as input. Post hoc pairwise comparisons, where available, were performed using Bonferroni correction. Similarly, proportions of the major and minor complications of the same sets of studies were compared to each other, with the application of Pearson’s chi^2 test of independence. Once more, post hoc pairwise comparisons, where available, were performed using Bonferroni correction. All statistical tests were two-sided, and statistical significance was defined as *p* < 0.05. Implementation was performed with R, the language for statistical computing, along with the RStudio IDE, both of which are well-known open-source products. More specifically, a set of functions from the base library were used and from the libraries “meta”, “metafor”, “dmetar”, “ggplot2”, and “binom”.

## Results

### Data extraction

Our database search following duplicate removal yielded a total of 630 records; 240 were removed after title review and 243 after abstract review. We retrieved the full texts of 147 manuscripts to screen them in their entirety; thirty-four studies were deemed appropriate for inclusion in our qualitative synthesis (Table 1). The most common reasons for the exclusion of full-text articles (113) were studies reporting on Neer type II fractures in general (*N* = 62) or only om IIA and V types (*N* = 10) and studies without distinct clinical outcomes and/or complication rates among Neer type IIA and IIB fractures (*n* = 30).

**Table 1:**
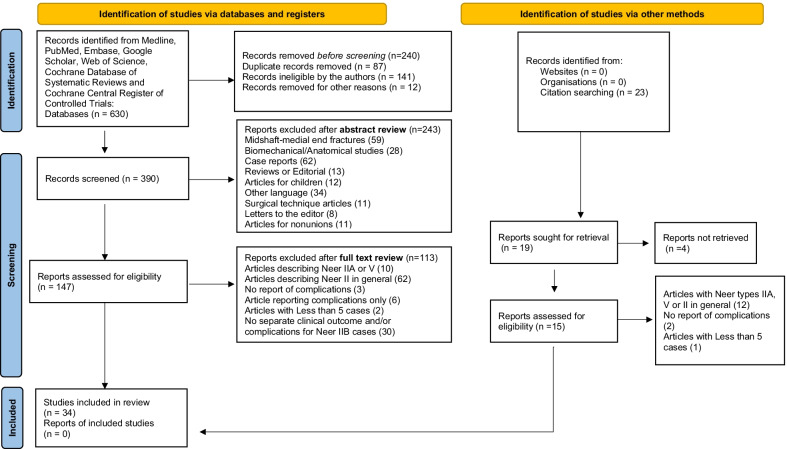
PRISMA work flow and eligibility of the studies

### Characteristics of included studies

The total number of retrieved patients with Neer type IIB (IIC) distal clavicle fractures was 790, ranging from 6 to 82 patients. The mean age of the patients was 40.1 years (30.3–49 years), the female percentage was 37%, and the mean follow-up period was 29.3 months (4.6–67 months). The key characteristics of the 34 included studies are summarized in Table 2.

**Table 2:**
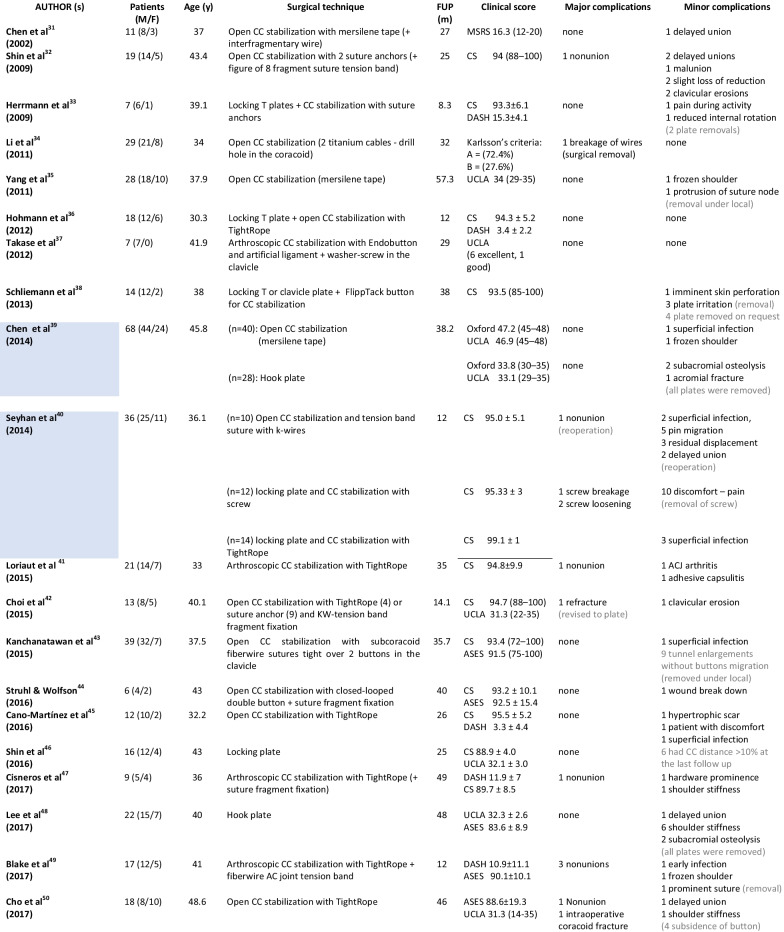

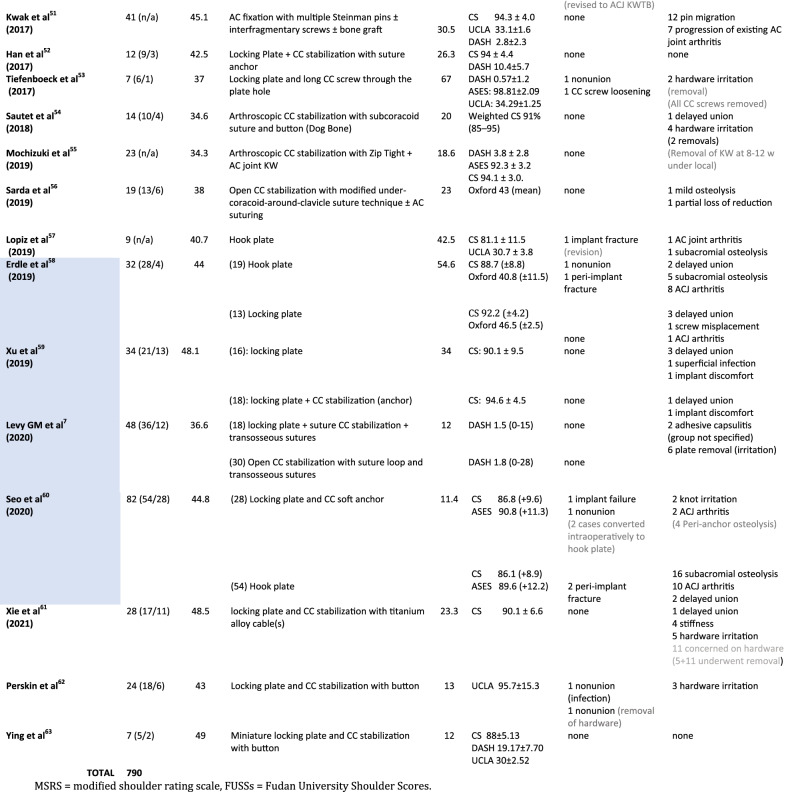
Overall characteristics of the included studies (in light blue are comparative studies)

MSRS, modified shoulder rating scale; FUSSs, Fudan University Shoulder Scores.

In total, 132 (16.7%) patients received a hook plate [39, 48, 57, 58, 60]; 252 (31.8%) received a locking plate [7, 18, 33, 36, 38, 40, 46, 52, 53, 58–62]; 368 (46.4%) received CC stabilization [7, 31, 32, 34, 35, 37, 39–45, 47, 49, 50, 54–56]; and 41 (5.1%) received transacromial transfixation [51]. In the locking plate group, the majority of patients (82%) had additional CC augmentation (CCA) with a) coracoid screws (7.5%); b) CC sutures, cables or anchors (44%); and c) CC buttons (30.5%). In the CC stabilization group, there were 91 arthroscopic procedures (49 with additional ACJ transfixation, 42 without) and 274 open procedures (108 with ACJ transfixation and 166 without). Finally, there were 6 retrospective comparative studies comparing (a) open CC stabilization with tape versus hook plate [39], (b) open CC stabilization + tension band suture + k-wires versus locking plate + CC stabilization with screw versus locking plate + CC stabilization with TightRope [40], (c) locking plate vs. hook plate [58], (d) locking plate versus locking plate + CC stabilization with anchor [59], (e) locking plate + suture CC stabilization + transosseous sutures vs. open CC stabilization with suture loop + transosseous sutures [7] and (f) locking plate + CC stabilization with soft anchor vs. hook plate [60].

### Risk of bias assessment

All studies were retrospective, with the majority of them having a fair MINORS score (Table 3). Only 2/28 noncomparative studies demonstrated an “excellent” score greater than 12 (max = 16). Among the 6 comparative studies, only one had an “excellent” score of 20 (max = 24). Thus, our systematic review had a low level of evidence (IV).

**Table 3:**
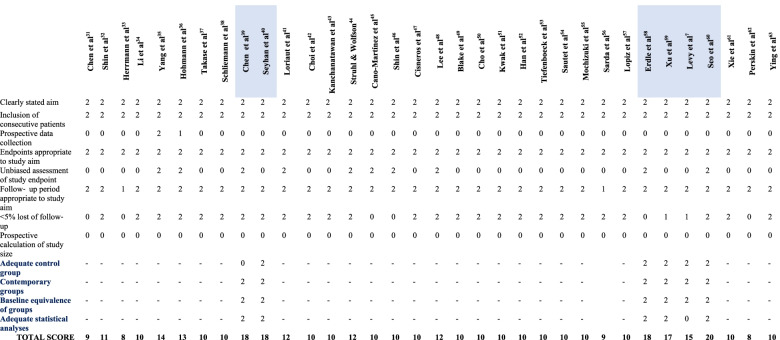
Risk of bias assessment of the included studies according to MINORS tool

Comparative studies are in blue

### Outcome scores

**Fig. 1:**
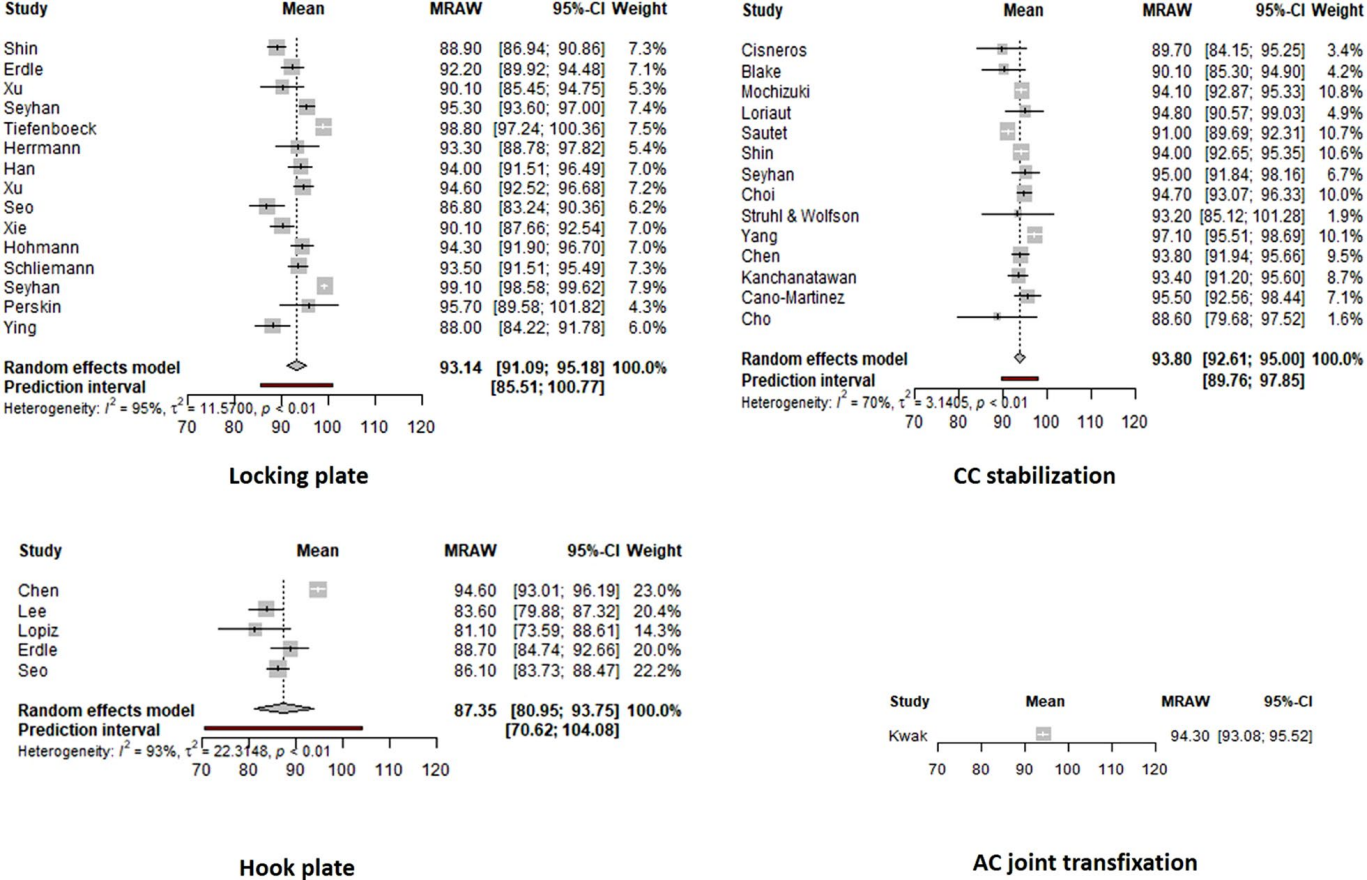
Forest plots of the overall clinical outcome for the four surgical techniques

Clinical results were reported with various clinical scores, including the Constant Score (27 studies), UCLA (13 studies), DASH (12 studies), ASES (8 studies), Oxford score (5 studies), Karlsson’s criteria (1 study) and Modified Shoulder Rating Scale for Clavicle Fractures (1 study). Twenty-three studies (67.7%) utilized more than one score for final clinical evaluation. A meta-analysis was performed that included all studies utilizing the Constant, ASES and UCLA scores (after transformation to a 100-scale). The forest plots of the four main techniques are presented in Fig. 1. Locking plate, CC stabilization and AC joint transfixation showed excellent clinical results in contrast to hook plate, which showed statistically significant lower clinical scores (*p* = 0.0009). The comparison of locking plate fixation with and without CC augmentation showed no difference (*p* = 0.3029); similarly, no difference was noticed among the 3 groups of locking plate augmentations (anchors, CC screw, button) [*p* = 0.7613]. Finally, the comparison between arthroscopic and open CC stabilization techniques showed a statistically important difference in favour of better clinical outcomes for open techniques (*p* = 0.0416).

### Complications

The overall major complication rate was 4.5% for the hook plate, 1.9% for the locking plate with CC augmentation and 2.4% for the CC stabilization group (Table 4). Interestingly, there were no major complications in the plate fixation without CC augmentation and ACJ transfixation groups. Regarding minor complications, the AC joint transfixation group demonstrated the highest complication rate (46%), followed by the hook plate group (42%), the locking plate group (23.8%) and the CC stabilization group (14.24%). Our meta-analysis did not show any significant difference among the four groups regarding the major complication rate (*p* = 0.3147). Additionally, there was no difference between the locking plate groups with and without CC augmentation (*p* = 0.6431) or among the 3 augmentation subgroups (*p* = 0.6563). Finally, there was no difference between open and arthroscopic CC stabilization techniques (*p* = 0.3271).

**Table 4:** Major and minor complications

Implant/surgical technique	*N* = 790	Major complications					Minor complications									
		***Non-union***	***Implant failure***	***Peri implant fracture***	***Deep infection***	***Coracoid or acromial fracture***	*Malunion – Delayed union*	*Hardware irritation, migration breakage, loosening*	*Subacromial osteolysis*	*Clavicular erosion*	*Button subsidence Peri-anchor or screw osteolysis*	*Slight Loss of reduction*	*AC joint arthrosis*	*Pain /discomfort* *Frozen shoulder /stiffness*	*Superficial infection*	*Scar problems, skin irritation* *wound breakdown*
**Hook plate** [39, 48, 57, 58, 60]	**132**	**1**		4		1	5		26				19	6		
**Locking plate**	**252**															
(−) CC augmentation [46, 58, 59]	45						6	2				6	1		1	
(+) CCA-screw [40, 53]	19	1						6						10		
(+) CCA-anchor(s), sutures, cables [7, 33, 52, 59–61]	111	1	1				2	8			4		2	8		
(+) CCA-button [18, 36, 38, 40, 62]	77	2						1							3	
**CC stabilization**	**365**															
Arthroscopic (91)																
(+) ACJ transfixation [47, 49, 55]	49	3						1						2	1	1
(−) ACJ transfixation [37, 41, 54]	42	1					1	4					1	1		
Open (274)																
(+) ACJ transfixation [7, 31, 32, 40, 42, 44, 56]	108	2		1			6	5		4		6			2	1
(−) ACJ transfixation [34, 35, 39, 43, 45, 50]	166	1		1			1	1						1	3	2
**AC joint trans-fixation** [51]	**41**							12					7			

**Fig. 2:**
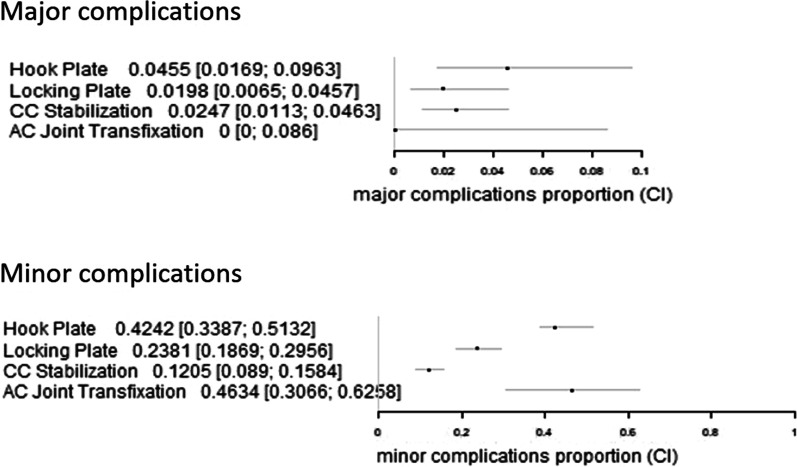
Forest plots of the overall major and minor complication rates

In contrast, there was a significant difference in the rate of minor complications in favour of CC stabilization in contrast to other techniques (*p* < 0.0001). There was no difference in the rate of minor complications between the plate fixation with and without augmentation groups (*p* = 0.0646) or between the open and arthroscopic CC stabilization groups (*p* = 0.8438). The forest plot of the complication rate is presented in Fig. 2.

## Discussion

The most important findings of the present systematic review on Neer type IIB (IIC) distal clavicle fractures are as follows: (a) the hook plate technique demonstrated lower functional scores in contrast to other techniques; (b) there was no difference in the rate of major complications among the four different techniques; (c) the CC augmentation of a standard locking plate with screws, sutures, cables, anchors or buttons did not improve clinical outcomes and provided a similar major complication rate, and the button augmentation subgroup had better results regarding minor complications; (d) open CC stabilization techniques showed better clinical outcomes than the arthroscopic techniques and same rate of major and minor complications; and (e) the AC joint transfixation technique had excellent early clinical outcomes and no major complications but the highest rate of minor complications (46%), although these results were extracted from only one study [51]. In this particular study, 7/41 (17%) patients had evidence of posttraumatic AC joint arthritis in a mean follow-up period of 30.5 months; this risk must be taken into account when AC transfixation is applied together with the possibility of KW breakage or migration (29%).

Our results are different from those the most recent systematic review by Uittenbogaard et al. (2021) [26] in 2284 patients, which included all Neer type II (IIA and IIB) distal clavicle fractures; according to these authors, the hook plate technique showed lower Constant scores than CC stabilization but no significant differences when the hook plate group was compared with the locking plate and tension band wire/K-wire groups. They also found higher Constant scores in those patients with supplemental CC fixation when locking plates were used, a finding that was not confirmed in our study.

Several other systematic reviews have also shown inconsistent results. Oh et al. [20] performed a systematic review in 365/425 surgically treated patients with Neer type II fractures (2011) before the wide use of locking plate fixation and found a higher complication rate with the use of the hook plate (40.7%) and tension band wiring (20.0%) than with coracoclavicular (4.8%), intramedullary (2.4%) and interfragmentary fixation (6.3%). Stegeman et al. [21] reported a meta-analysis of 21 studies including 350 patients (2013) with type II distal clavicle fractures; union was achieved in 98% of the patients, and the functional outcomes were similar among the treatment modalities (hook plate, different other plates, CC sutures, screws and Knowles pins). In this study, hook plate fixation was associated with an 11-fold increased risk of major complications compared to intramedullary fixation and a 24-fold increased risk compared to suture anchoring. Boonard et al. [22] performed a systematic review and network meta-analysis in 2018; among ten comparative studies (*n* = 505 patients) and one RCT study (*n* = 42), the Constant-Murley scores of coracoclavicular fixation were significantly higher than those of hook plate and tension band wiring. Asadollahi and Bucknill (2019) [23], in a systematic review of eleven comparative studies to hook plate fixation, including 634 patients, found no significant difference between the functional outcome and union rate between hook plate fixation, CC stabilization and locking plate fixation. Hook plate fixation resulted in a higher Constant score than tension band wiring (TBW) but was also associated with a higher complication rate than CC stabilization and the locking plate. Vannabouathong et al. [24] reported that in type II distal clavicle fractures, a locking plate with or without CC suturing yielded significantly better outcomes than K-wires with or without tension bands, CC suturing alone, a locking plate with a CC screw, a hook plate, and a sling. In 2021, Malik et al. [26] and Yagnik et al. [28] systematically reviewed the reports of arthroscopic or arthroscopically assisted CC stabilization techniques; despite overall good to excellent shoulder function in both reports, the first one found considerably lower union rates up to 70% and overall complication rates as high as 28.6%, and the second reported union rates in 94.1% of the fractures and overall complication rates of 27.4%, of which 12% were considered major complications, and only 6% required a reoperation for hardware-related complications. Finally, Panagopoulos et al. [27] systematically reviewed the safety and efficacy of CC stabilization techniques (67% open, and 33% arthroscopic) in 2021, particularly in Neer type IIB fractures, and found very good to excellent clinical scores and overall major and minor complication rates of 2.6% and 12.8%, respectively. Major complications were more frequent with arthroscopic-assisted techniques (4.3%) than with open techniques (1.8%).

From all these systematic reviews, it is clear that hook plate fixation is associated with more major complications and worse clinical results and should likely no longer be considered as an ideal implant for Neer type IIB distal clavicle fractures. In our meta-analysis, we found a similar rate of major complications to the other techniques but a significant difference in the rate of minor complications. The other techniques demonstrated similar clinical outcomes, high union rates and lower rates of complications, but we do not yet have a precise answer to whether CC augmentation to a standard locking plate is always necessary or whether arthroscopic CC stabilization with buttons is superior to the classic open technique. Nevertheless, the current trend in the literature is plate fixation with CC augmentation and open or arthroscopic CC stabilization techniques. A critical advantage of arthroscopic-assisted stabilization is the ability to recognize and address any concomitant intra-articular shoulder pathology during diagnostic arthroscopy. A recent study from Marín Fermín et al. [63] revealed a mean of 17.70% concomitant glenohumeral injuries, whereas 84.21% of them required additional surgical management; rotator cuff injuries, labral tears, and biceps pulley lesions were the most common concomitant injuries.

One of the strengths of our study is that it constitutes the first systematic review in the literature that analyses only Neer type IIB fractures of the distal clavicle. We included 34 reports with 790 patients, one of the largest populations even reported, and we were able to show that almost 80% of the cases were treated either with a locking plate (simple or augmented) or CC stabilization (open or arthroscopic). This shows a clear trend in the literature over the last 10 years towards the abandonment of the hook plate and AC joint transfixation techniques. On the other hand, our study has several limitations: (a) the discrepancies in the data available from the included studies and mainly the heterogeneity of fracture patterns as most of the studies did not subclassify the type II (A and B) (it is of major importance that > 100 studies were excluded from this review for that reason); (b) the low level of evidence of the included studies, as there were no randomized comparative or prospective studies; (c) discrepancies also existed in reported outcome measures, as follow-up was obtained at various time points and the evaluation was made with different and sometimes not shoulder-specific scores (DASH) (which, for example, can underestimate the incidence of AC joint arthritis in the long term); and d) the number of the included patients in these studies was relatively low.

## Conclusions

Our systematic review and meta-analysis of the best available surgical technique for the treatment of Neer type IIB (IIC) distal clavicle fractures showed great heterogeneity among studies, a low level of evidence, inconsistent classification methods, and a lack of appropriate outcome evaluation and reporting of complications. While hook plate fixation seems to demonstrate inferior clinical results with a high rate of minor complications, locking plate fixation with or without cortical button augmentation and open CC stabilization showed the best results. More prospective comparative studies are needed to increase the existing level of evidence; such studies must include a large number of patients with proper subclassification of different Neer types, incidence of AC joint arthritis and detailed reports of major and minor complications.

## Data Availability

The datasets used and/or analyzed during the current study are available from the corresponding author on reasonable request.

## Abbreviations

MINORS: Methodological Index for Non-Randomized Studies
CC: Coracoclavicular
ACJ: Acromioclavicular joint
TBW: Tension band wiring
RCTs: Randomized controlled trials
CCs: Coracoclavicular Stabilization
KW: Kirshner wire
LPF: Locking Plate Fixation
HPF: Hook Plate Fixation
ACJTF: AC joint trans-fixation
CS: Constant Score
ASES: American Shoulder and Elbow Surgeons Shoulder score
UCLA: University of California at Los Angeles Shoulder Score
